# Supplementary figures and images for: Lineage-informative microhaplotypes for spatio-temporal surveillance of Plasmodium vivax malaria parasites
Source: medRxiv. 2023 Mar 16:2023.03.13.23287179. Preprint. [Version 1] doi: 10.1101/2023.03.13.23287179 (PMC10055443; doi:10.1101/2023.03.13.23287179)

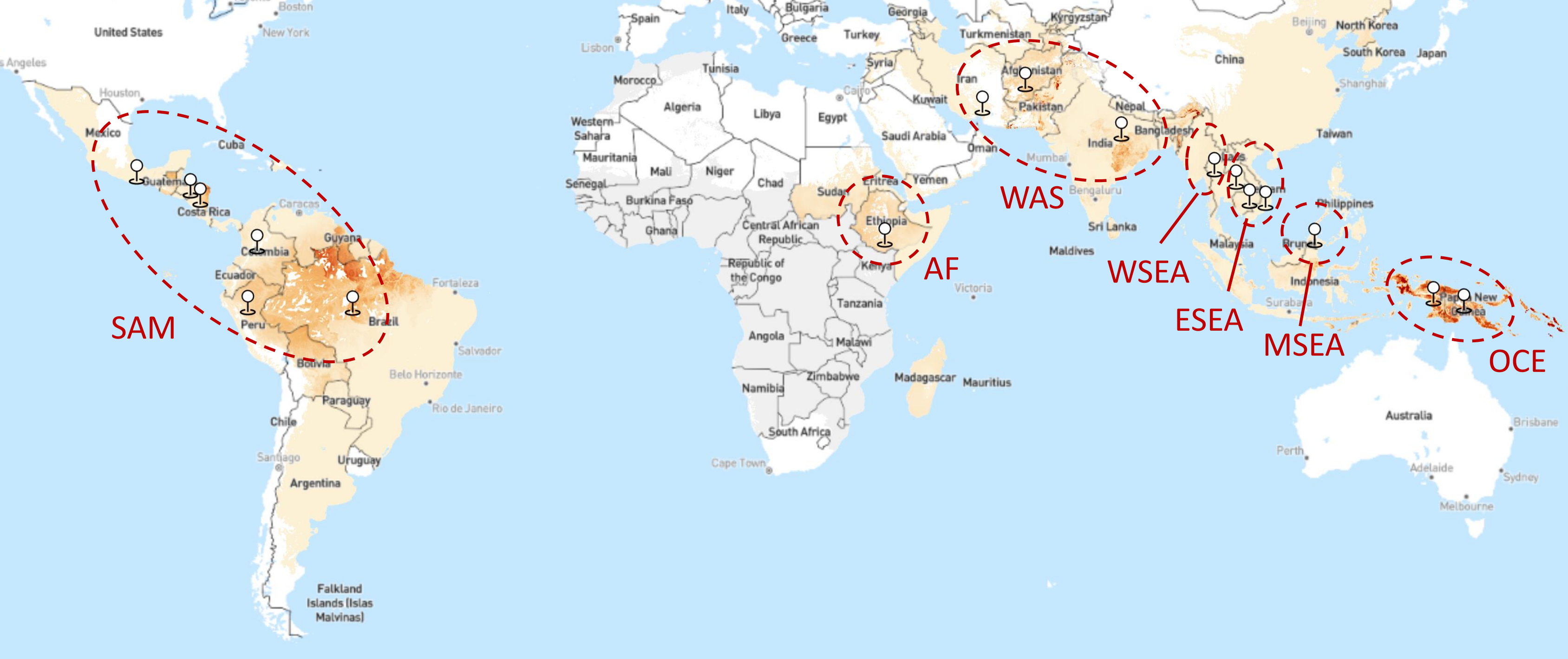

Supplement: Supplement 3 [file media-3.tif]

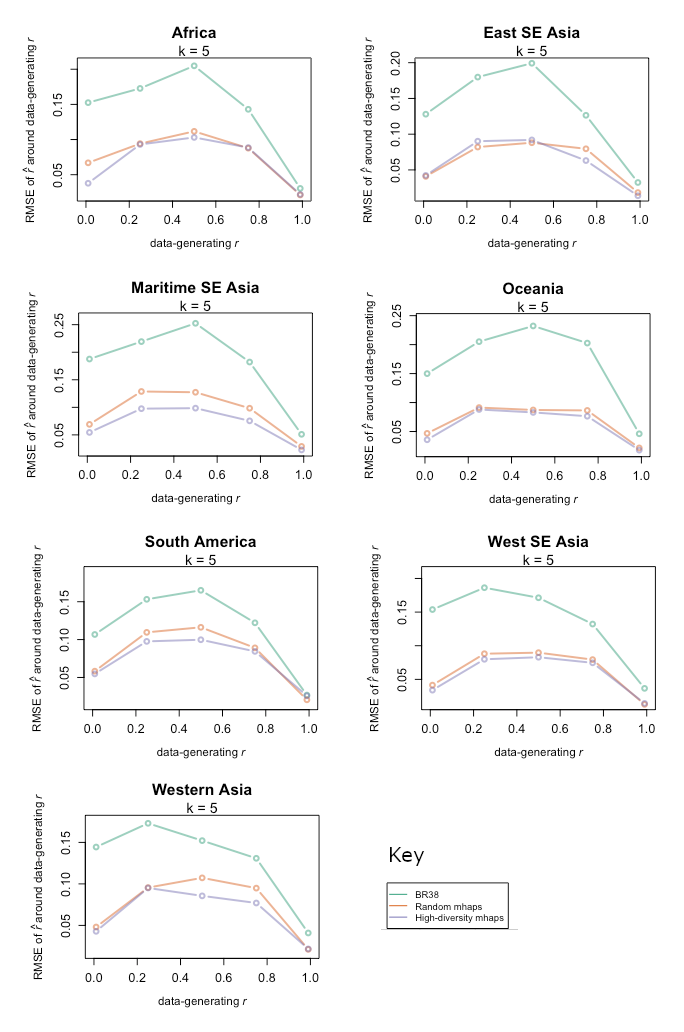

Supplement: Supplement 4 [file media-4.tif]

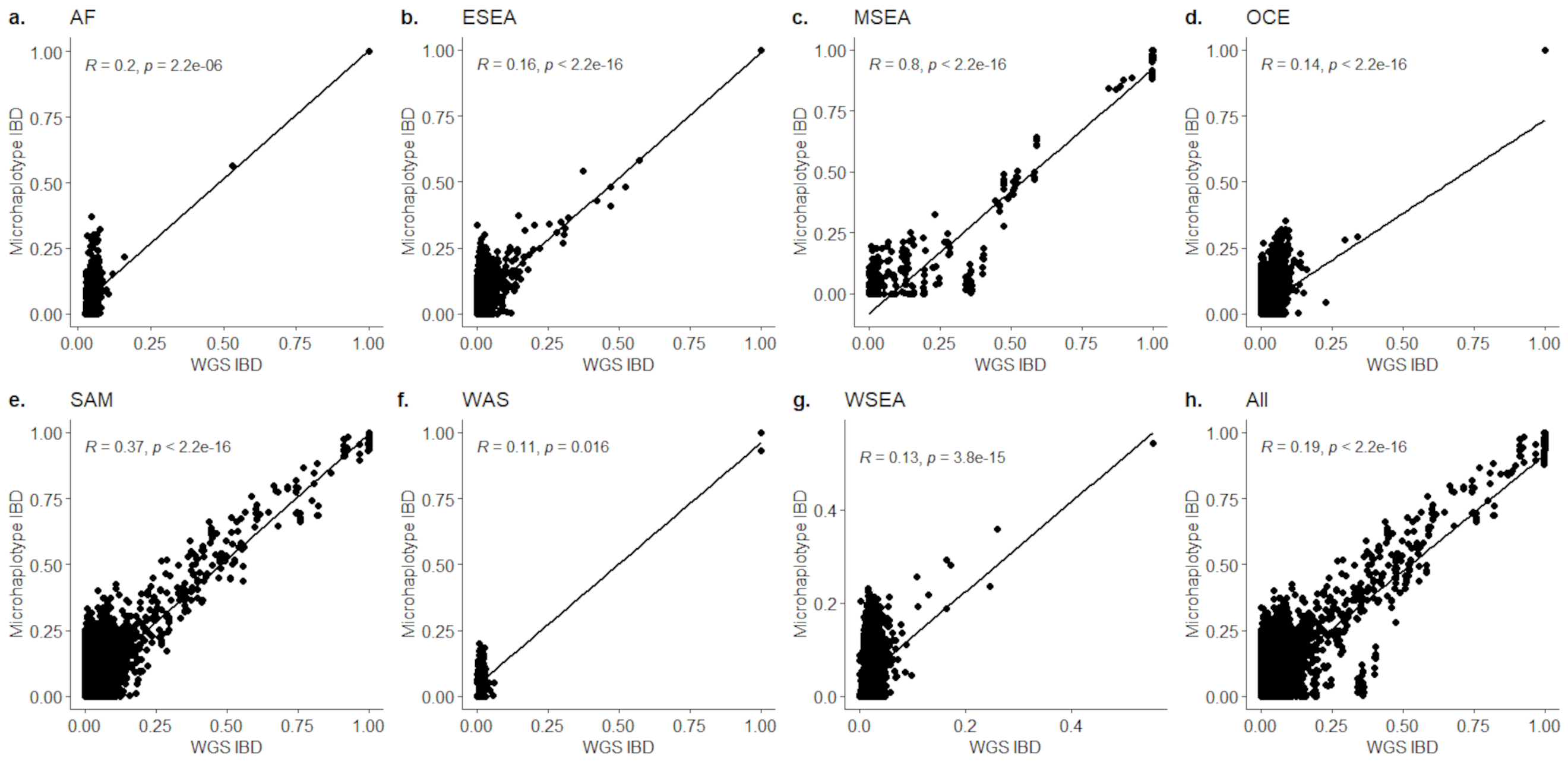

Supplement: Supplement 5 [file media-5.tif]

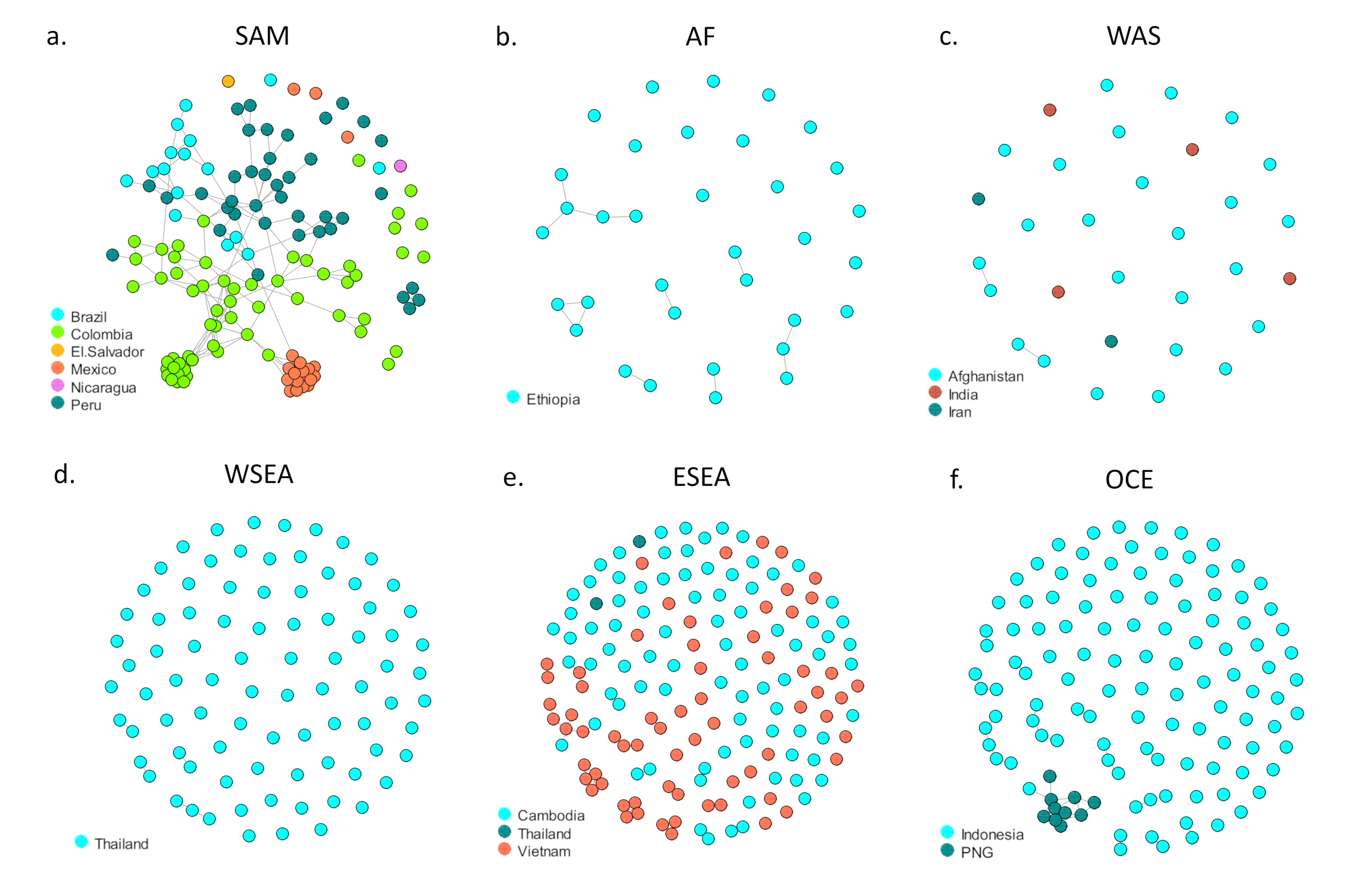

Supplement: Supplement 6 [file media-6.tif]
