# Supplementary material for: Lineage-informative microhaplotypes for spatio-temporal surveillance of Plasmodium vivax malaria parasites
Source: medRxiv. 2023 Mar 16:2023.03.13.23287179. Preprint. [Version 1] doi: 10.1101/2023.03.13.23287179 (PMC10055443; doi:10.1101/2023.03.13.23287179)
Supplement: 7 [file NIHPP2023.03.13.23287179v1-supplement-7.pdf]

medRxiv preprint doi: https://doi.org/10.1101/2023.03.13.23287179; this version posted March 16, 2023. The copyright holder for this preprint (which was not certified by peer review) is the author/funder, who has granted medRxiv a license to display the preprint in perpetuity.
It is made available under a CC-BY 4.0 International license .

Supplementary Material

Supplementary Table 1. Microhaplotype panel marker selection information.

Region	Chromosome: coordinate: SNP position	Effective cardinality	Heterozygosity
AF	PvP01_14_v1:3009931:19,27,72,78,81,89,119,139,150	18.94	0.95
AF	PvP01_07_v1:78144:4,11,28,79,127,130,136,156	10.25	0.9
AF	*PvP01_05_v1:1369384:7,52,99,114,121,126,136,164	9.62	0.9
ESEA	*PvP01_05_v1:1369384:7,52,99,114,121,126,136,164	21.28	0.95
ESEA	PvP01_01_v1:772094:42,60,64,65,117	12.97	0.92
ESEA	PvP01_07_v1:78144:4,11,28,79,127,130,136,156	10.69	0.91
MSEA	*PvP01_05_v1:1369384:7,52,99,114,121,126,136,164	23.91	0.96
MSEA	PvP01_11_v1:1423352:18,64,79,126,137,160,167	15.24	0.93
MSEA	PvP01_14_v1:2261605:80,96,114,135,189	11.9	0.92
OCE	*PvP01_05_v1:1369384:7,52,99,114,121,126,136,164	26.05	0.96
OCE	PvP01_11_v1:1423352:18,64,79,126,137,160,167	17.93	0.94

medRxiv preprint doi: https://doi.org/10.1101/2023.03.13.23287179; this version posted March 16, 2023. The copyright holder for this preprint (which was not certified by peer review) is the author/funder, who has granted medRxiv a license to display the preprint in perpetuity.
It is made available under a CC-BY 4.0 International license .

OCE	PvP01_06_v1:286251:5,6,28,75,168,179,184	13.64	0.93
SAM	*PvP01_05_v1:1369384:7,52,99,114,121,126,136,164	27.16	0.96
SAM	PvP01_11_v1:1423352:18,64,79,126,137,160,167	19.94	0.95
SAM	PvP01_06_v1:286251:5,6,28,75,168,179,184	16.57	0.94
WAS	*PvP01_05_v1:1369384:7,52,99,114,121,126,136,164	11.72	0.91
WAS	PvP01_06_v1:983158:17,19,24,35,117,136,175	10.74	0.91
WAS	PvP01_06_v1:286251:5,6,28,75,168,179,184	8.54	0.88
WSEA	*PvP01_05_v1:1369384:7,52,99,114,121,126,136,164	36.9	0.97
WSEA	PvP01_11_v1:1423352:18,64,79,126,137,160,167	24.2	0.96
WSEA	PvP01_06_v1:286251:5,6,28,75,168,179,184	15.39	0.94

Supplementary Table 2. Properties of the three most diverse markers in each geographic region for the High-diversity microhaplotype panel. *The PvP01_05_v1:1369384:7,52,99,114,121,126,136,164 microhaplotype was one of the top three most diverse markers in all seven geographic regions.

medRxiv preprint doi: https://doi.org/10.1101/2023.03.13.23287179; this version posted March 16, 2023. The copyright holder for this preprint (which was not certified by peer review) is the author/funder, who has granted medRxiv a license to display the preprint in perpetuity.
It is made available under a CC-BY 4.0 International license .

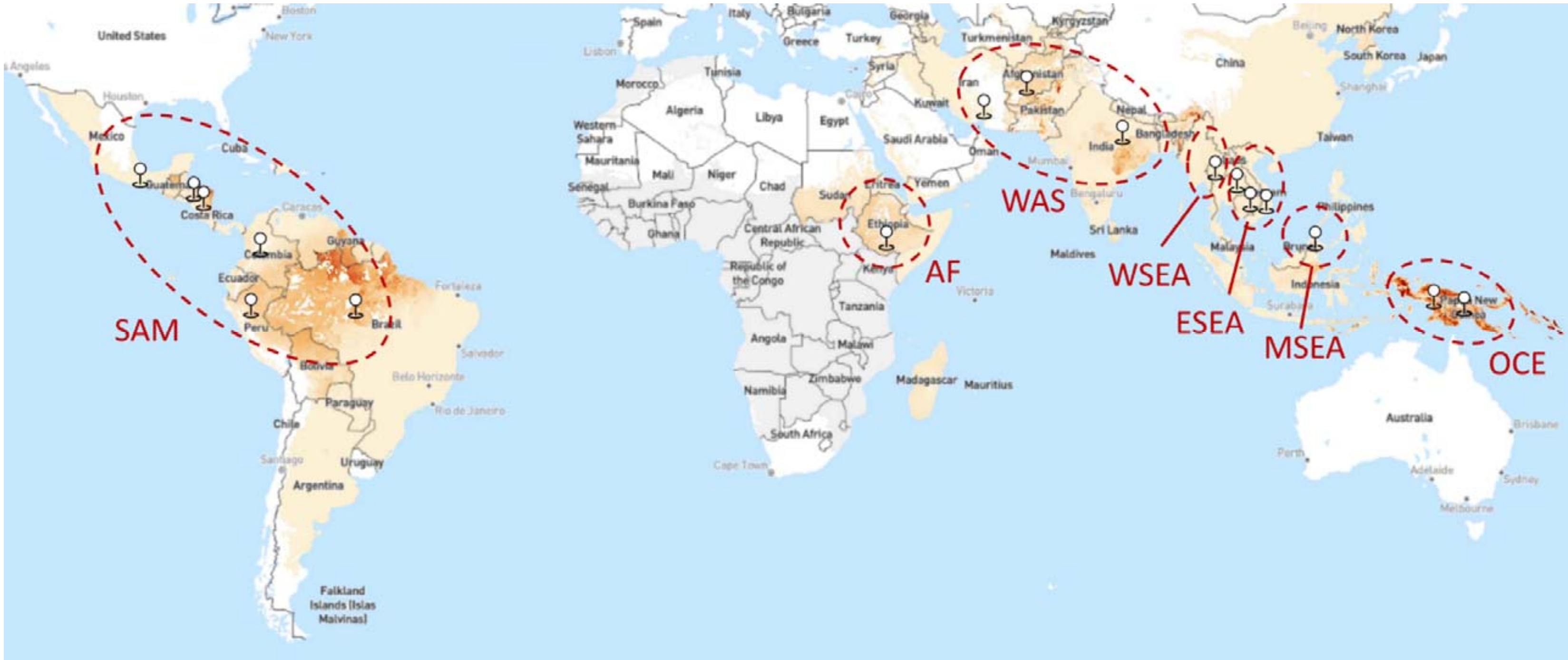

Supplementary Figure 1. *P. vivax* incidence map illustrating regional country groupings. The baseline *P. vivax* incidence map was derived from the Malaria Atlas Proj (MAP) and presents the number of newly diagnosed *P. vivax* cases per 1,000 population in 2020[41]. The labelled, dashed red lines indicate the boundaries of the geographic regions included in the identity by descent (IBD) analyses: SAM (South America), AF (Africa), WAS (West Asia), WSEA (West Southeast Asia), ESEA (East Southeast Asia), MSEA (Maritime Southeast Asia) and OCE (Oceania). Pinpoints indicate the countries included in each regional grouping.

medRxiv preprint doi: https://doi.org/10.1101/2023.03.13.23287179; this version posted March 16, 2023. The copyright holder for this preprint (which was not certified by peer review) is the author/funder, who has granted medRxiv a license to display the preprint in perpetuity.
It is made available under a CC-BY 4.0 International license .

medRxiv preprint doi: https://doi.org/10.1101/2023.03.13.23287179; this version posted March 16, 2023. The copyright holder for this preprint (which was not certified by peer review) is the author/funder, who has granted medRxiv a license to display the preprint in perpetuity.
It is made available under a CC-BY 4.0 International license .

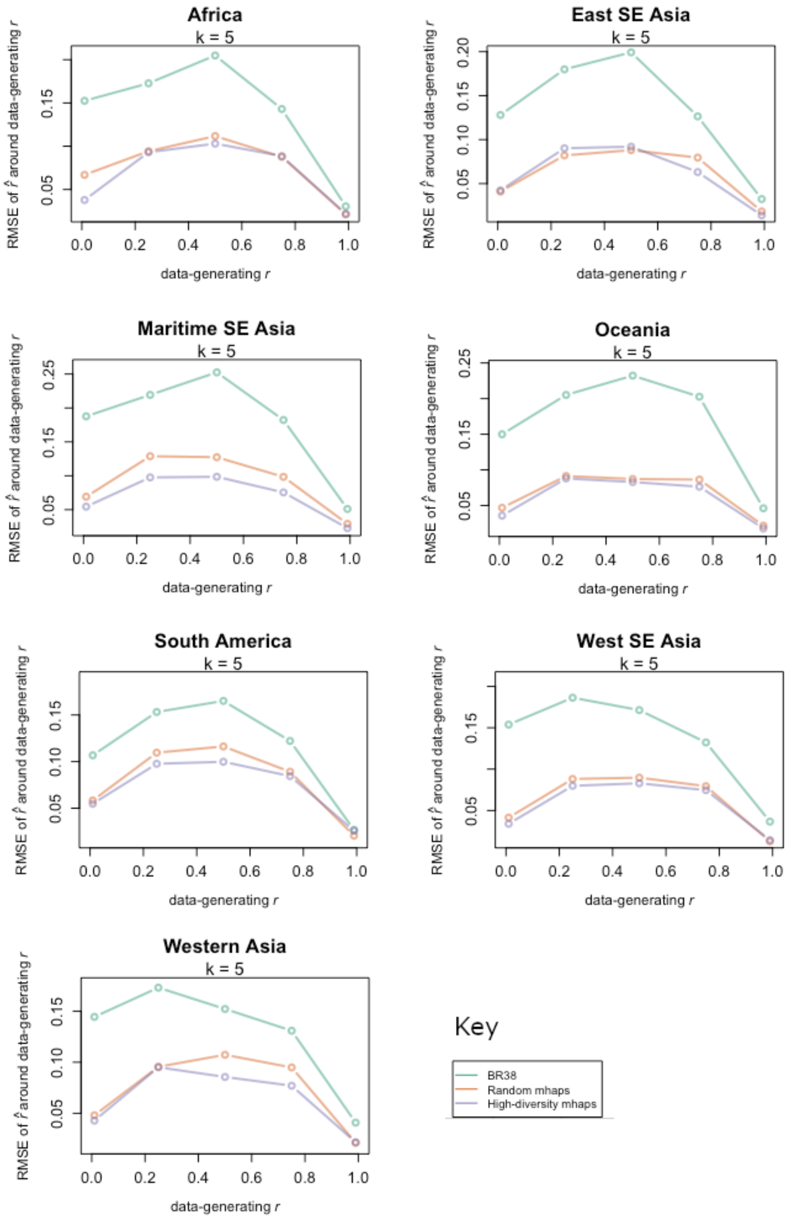

Supplementary Figure 2. Comparative accuracy in relatedness prediction at Random and High-diversity microhaplotype panels, and the 38-SNP Broad barcode. Root mean square error (RMSE) of relatedness estimates based on data simulated using various data-generating relatedness and switch rate parameters, *r* and *k*, respectively. Data are presented on 3 marker panels: High-diversity SNP microhaplotype panel, Random-SNP microhaplotype panel and 38 Broad barcode biallelic SNPs. Panel comparisons are presented by geographic region; AF (Africa), ESEA (East Southeast Asia), MSEA (Maritime Southeast Asia), OCE (Oceania), SAM (South America), WAS (West Asia) and WSEA (West Southeast Asia).

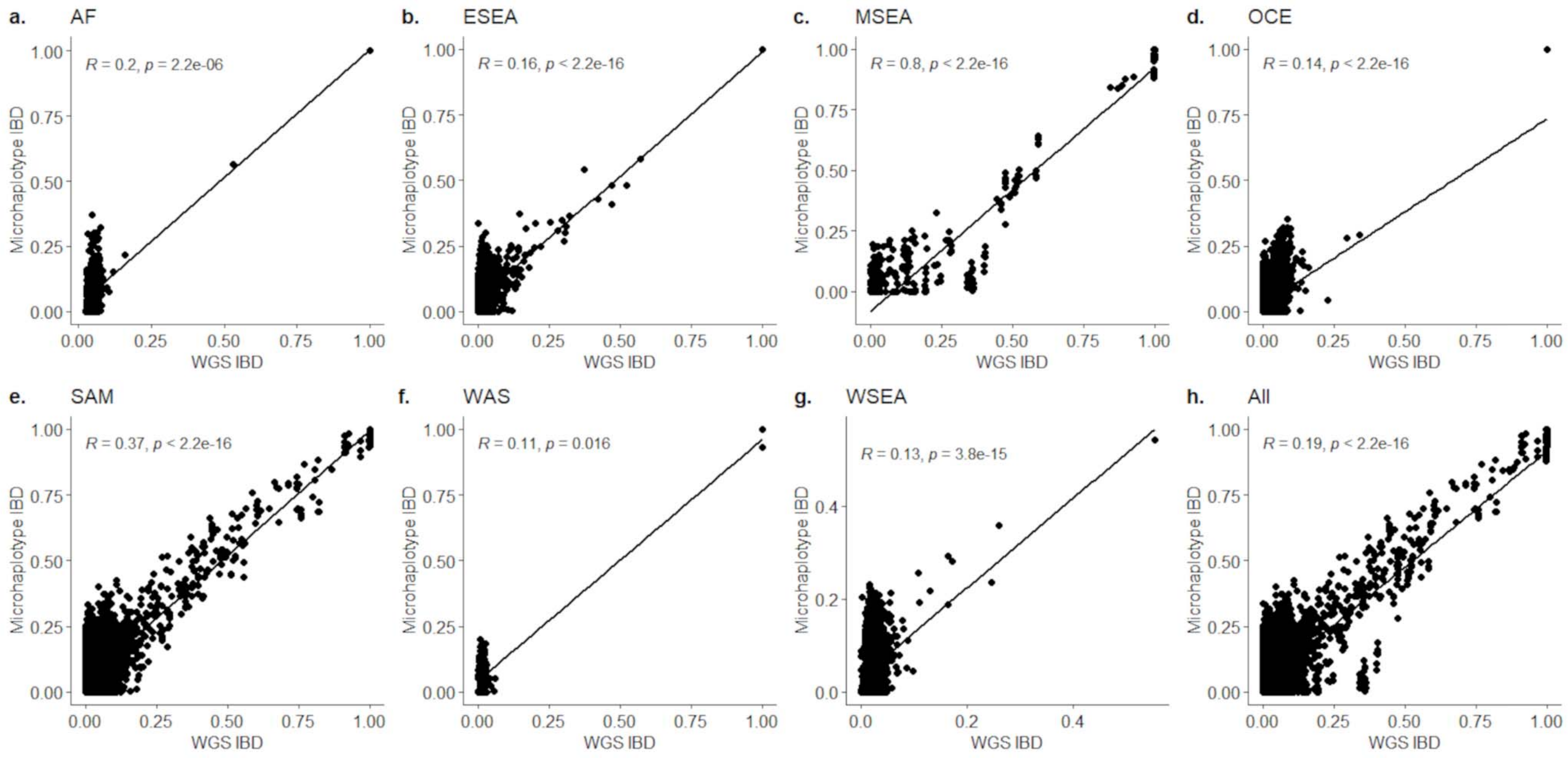

Supplementary Figure 3. Correlations between microhaplotype and genomic estimates of IBD in the regional datasets. Panels a) to g) represent Africa (AF), East Southeast Asia (ESEA), Maritime Southeast Asia (MSEA), Oceania (OCE), South America (SAM), West Asia (WAS), and West Southeast Asia (WSEA). The microhaplotyp and whole genome sequence (WGS) IBD estimates reflect pairwise estimates at the High-diversity SNP microhaplotype panel and a set of 898,448 genome-wide SNPs calculations were performed using *hmmIBD* on the 615 monoclonal sample set. Correlations were assessed with Spearman's rho statistic (using a paired test) and presented with the associated p-value. At an alpha of 0.05, significantly positive correlations were observed in all regions.

medRxiv preprint doi: https://doi.org/10.1101/2023.03.13.23287179; this version posted March 16, 2023. The copyright holder for this preprint (which was not certified by peer review) is the author/funder, who has granted medRxiv a license to display the preprint in perpetuity.
It is made available under a CC-BY 4.0 International license .

medRxiv preprint doi: https://doi.org/10.1101/2023.03.13.23287179; this version posted March 16, 2023. The copyright holder for this preprint (which was not certified by peer review) is the author/funder, who has granted medRxiv a license to display the preprint in perpetuity.
It is made available under a CC-BY 4.0 International license .

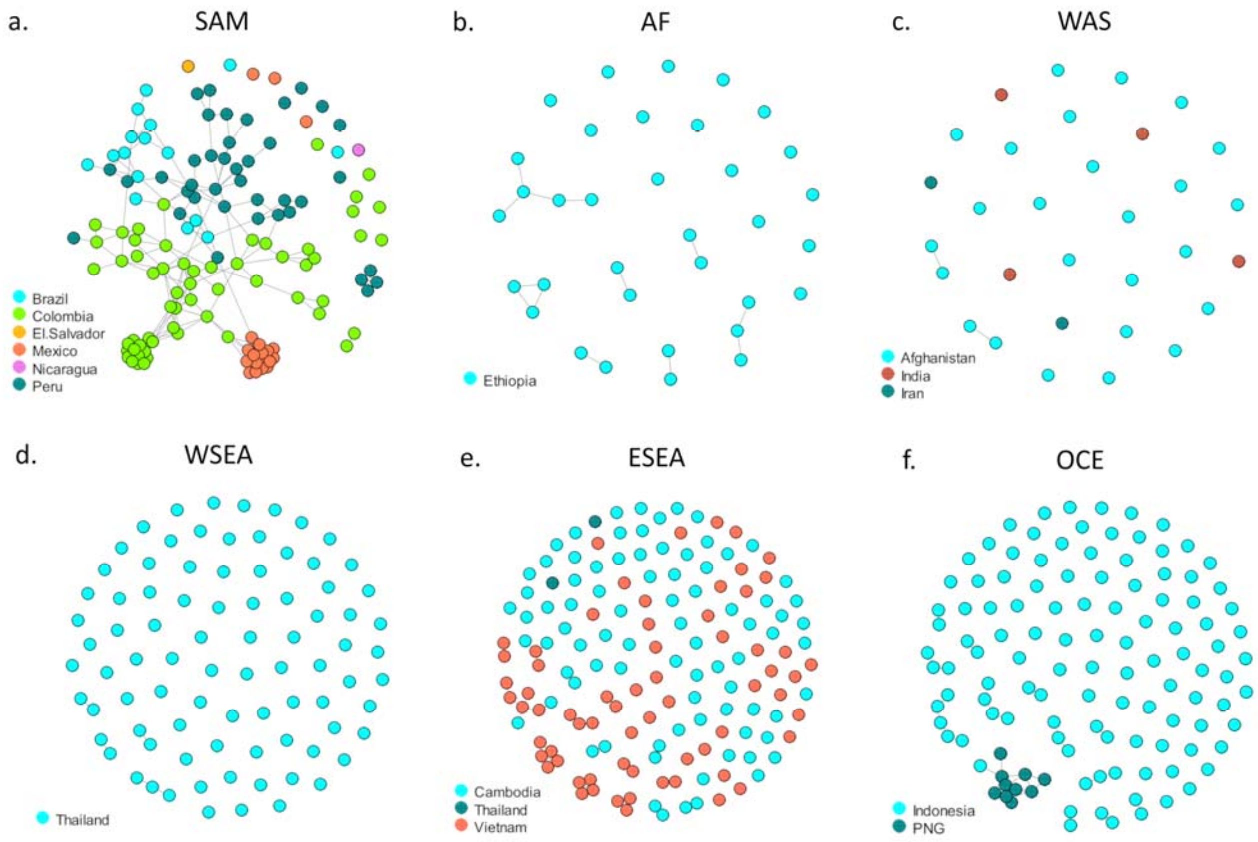

Supplementary Figure 4. Regional microhaplotype-based infection networks. Panels a) to e) represent South America (SAM), Africa (AF), West Asia (WAS), West Southeast Asia (WSEA), East Southeast Asia (ESEA) and Oceania (OCE). The networks were generated from the High-diversity SNP microhaplotype panel in the 615 monoclonal sample set. The isolates from Maritime Southeast Asia (MSEA) were mostly from Malaysia (57/59) and are represented in Figure 5. Each circle reflects an infection, colour-coded by country, and line lengths reflect relatedness (shorter lines reflect greater relatedness) at a connectivity threshold of minimum identity by descent (IBD) 0.25 (half-siblings or greater relatedness). Each circle reflects an infection, with colour-coding by country.
